# Endoscopic Ultrasound-Guided Celiac Plexus Neurolysis in Pancreatic Cancer: A Prospective Pilot Study of Safety Using 10 mL versus 20 mL Alcohol

**DOI:** 10.1155/2013/327036

**Published:** 2013-01-08

**Authors:** Julia K. LeBlanc, Susan Rawl, Michelle Juan, Cynthia Johnson, Kurt Kroenke, Lee McHenry, Stuart Sherman, Kathy McGreevy, Mohammad Al-Haddad, John DeWitt

**Affiliations:** ^1^Division of Gastroenterology and Hepatology, Indiana University Medical Center, 550 North University Boulevard, UH 4100, Indianapolis, IN 46202, USA; ^2^Department of Adult Health, School of Nursing, Indiana University, Indianapolis, IN 46202, USA; ^3^Department of Biostatistics, School of Medicine, Indiana University, 410 W. Tenth Sreet, Suite 3000, Indianapolis, IN 46202-3012, USA; ^4^School of Medicine, Indiana University, Indianapolis, IN 46202-3082, USA; ^5^Regenstrief Institute, Indianapolis, IN, USA; ^6^Houston VA Health Services Research & Development Center of Excellence, Indianapolis, IN, USA

## Abstract

*Background*. The dose of alcohol used in EUS-CPN is not standardized. The objective was to compare the safety of 20 mL alcohol versus 10 mL alcohol during EUS-CPN for patients with pancreatic cancer-related pain. *Methods*. 20 patients were selected to receive 10 mL or 20 mL of alcohol during EUS-CPN. Followup was done at baseline, 24 hours, and weekly. Health-related quality of life (HRQoL) was assessed at baseline, week 2, week 4, and every 4 weeks thereafter until pain returned. *Results*. There were no major complications in both groups. Minor self-limited adverse effects were seen in 6 (30%) subjects and included lightheadedness in 1 (5%), transient diarrhea in 2 (10%), and transient nausea and vomiting in 3. Pain relief was similar in both groups: 80% in the 10 mL group and 100% in the 20 mL group (*P* = 0.21). The mean (± SD) duration of pain relief in the 10 mL and 20 mL groups was 7.9 ± 10.8 and 8.4 ± 9.2 weeks, respectively. 30% of patients in each group had complete pain relief. *Conclusions*. EUS-CPN using 20 mL of alcohol is safe. Similar clinical outcomes were seen in both groups. Further investigations to confirm these findings are warranted.

## 1. Introduction

One of the main concerns of patients with pancreatic cancer is pain [1]. For patients with pancreatic cancer, pain has a negative impact on quality of life (HRQoL) [2]. Pain will be present in a third of patients at the time of diagnosis, 30% to 50% undergoing treatment, and up to 90% with advanced disease [3–10]. After surgery for pancreatic cancer from 60% to 84% of patients reported moderate-to-severe pain [11]. While opioids are commonly used to relieve pain, their adverse side effects such as sedation, constipation, nausea, and vomiting have a negative impact on quality of life [3]. Celiac plexus neurolysis is not associated with these adverse effects and may improve survival among unresectable pancreatic cancer patients [5]. Although endoscopic ultrasound-guided celiac plexus neurolysis (EUS-CPN) does not have the side effects of opioids, it is not free of risks [12, 13]. Acute spinal cord infarction has been reported after EUS-CPN [13]. 

The amount of alcohol used in EUS-CPN ranges from 2 to 20 mL of alcohol [14–18]. There are no randomized studies that compare the safety of varying amounts of alcohol in celiac plexus neurolysis. We hypothesized that 20 mL of alcohol was safe in EUS-CPN. Onset of HRQoL and survival were also examined. 

## 2. Methods

This study was approved by the Institutional Review Board at Indiana University Medical Center. Consecutive patients with known or suspected unresectable pancreatic cancer and pain were enrolled. Written informed consent to participate in this study was obtained from all patients enrolled. Patients were excluded if they had the following: previous CPN (endoscopic or percutaneous), an implanted pain relieving device, or an arterial abdominal aneurysm. Patients were selected to receive 20 mL of 0.75% bupivacaine followed by 10 mL or 20 mL of alcohol for EUS-CPN. Opioid use was not a prerequisite for entry into this study. Pain relief was defined as a pain score less than or equal to 4, or at least a 30% decrease in the pain score compared to baseline pain without an increase in pain medication usage. 

### 2.1. EUS-CPN Procedure

**Figure 1:**
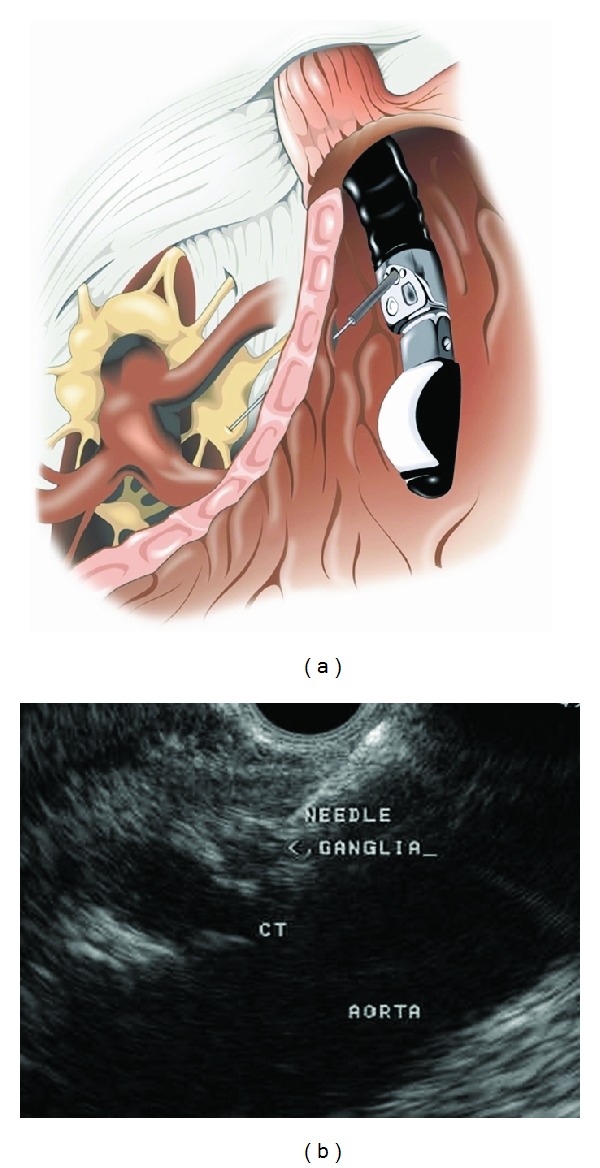
(a) EUS-CPN with direct injection into the celiac ganglia. (b) EUS image of direct celiac ganglia injection.

EUS-CPN is defined as injection of a neurolytic agent into the celiac plexus area or directly into celiac ganglia. After providing informed consent, subjects were sedated with intravenous fentanyl, midazolam, and/or Propofol. A 22-gauge hollow Echotip Ultra needle (Cook Medical, Winston-Salem, NC, USA) was passed through the working channel of the echoendoscope, through the posterior wall of the stomach and directly into the celiac ganglia (Figures 1(a) and 1(b)) when visualized or anterior to the celiac trunk. A 5 mL sterile saline-filled syringe was loaded onto the needle and was used to “test” the position of the needle tip. Through the needle 20 mL of 0.75% bupivacaine was injected followed by 10 mL or 20 mL of 98% alcohol. If celiac ganglia were visualized, they were directly injected until the ganglia borders were blurred. When celiac ganglia were not sonographically identified, the needle was targeted to the origin of the celiac artery takeoff within 2 to 3 mm. The medication is administered in the same sequential fashion. After the EUS-CPN procedure, each patient was observed for at least one hour in the recovery room. Each patient received 1000 mL of intravenous fluids and antibiotics. Oral antibiotics were prescribed for three days. Prior to discharge each patient was assessed by the physician. 

### 2.2. Followup

Baseline pain medication use and pain scores were documented for each patient. Each patient was called at 24 hours and weekly by a blinded research coordinator. Followup continued until the patient reported a return of their pain to baseline or died. Patients rated pain using a numeric rating scale (NRS) that ranges from 0 to 10 where 0 is “no pain” and 10 is “worst pain.” During each phone interview, patients were also asked to quantify their use of pain medications. During each phone interview, the subject's pain was assessed using the brief pain inventory (BPI). The BPI is a three question survey that rates intensity of pain (severity) and interference of pain with mood, physical activity, work, social activity, relations with others, sleep, and enjoyment of life [19, 20]. Two HRQoL instruments, EORTC QLQ-30 and EORTC PAN-26, were administered during phone interviews at baseline, week 2, week 4, and every 4 weeks thereafter until the subject reported a return of their pain to baseline or died. The date of death was documented for each subject.

### 2.3. Statistical Methods

The frequency of complications was calculated. Complete response was defined as a pain score of zero without an increase in pain medication usage. Kaplan-Meier estimates of duration of pain relief were calculated. Health-related quality of life (HRQoL) scores were summarized for each patient. Patients who died while still experiencing pain relief are censored from our calculation of pain-free survival. Patients who did not achieve pain relief within the first four weeks of followup are assigned zero for duration of pain relief and were not be censored. The overall survival is calculated from the time of EUS-CPN. 

## 3. Results

**Figure 2:**
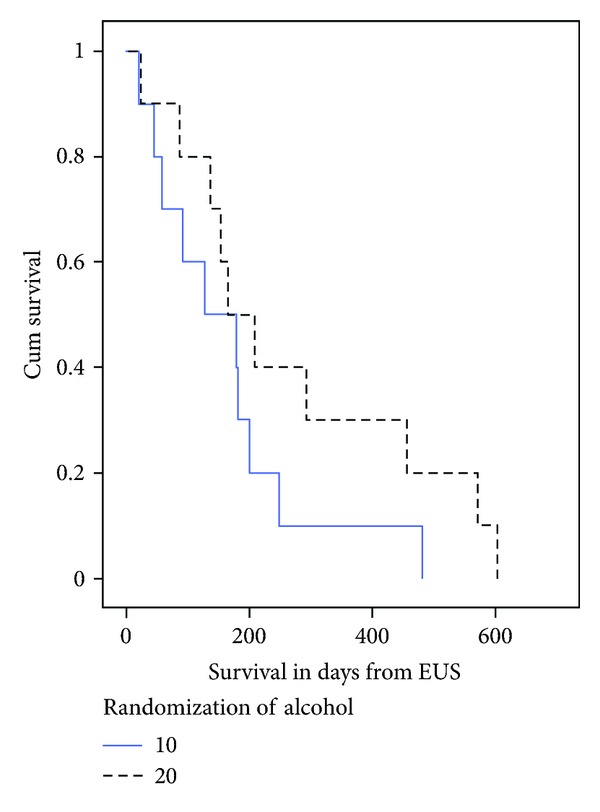
Survival in subjects by randomization. The survival curve shows the length of survival in days from the EUS-CPN procedure. The slashed line represents subjects in the 20 mL alcohol group, and the straight line represents subjects in the 10 mL alcohol group.

Patient characteristics are demonstrated in Table 1. The characteristics of pain relief in each group are demonstrated in Table 2. Among patients who received adjuvant chemotherapy and/or radiation, the median (range) duration of pain relief was 2.6 (0–32) weeks. Among patients who did not receive adjuvant chemotherapy and/or radiation the median (range) duration of pain relief was 2 (0–32) weeks. Pain scores are shown in Table 3. Four of 20 (20%) subjects (2 in each group) had pain relief that lasted over 16 weeks. Characteristics of complete responders (patients who had complete pain relief) are shown in Table 4. There were no deaths during the phone interview followup period of this study. Among the 10 patients who had direct injection of celiac ganglia, the median (range) duration of pain relief was 3 (0–32) and 3 (2–30) weeks for the 10 mL and 20 mL groups, respectively, (*P* = 0.69). In patients who *did not* have direct injection of celiac ganglia, the median (range) duration of pain relief 3 (2–8) and 1 (1–14) for the 10 mL and 20 mL alcohol groups, respectively, (*P* = 0.19). A Kaplan Meier survival curve is shown in Figure 2. 

### 3.1. Quality of Life

HRQoL at the week 4 of followup is shown in Table 5. Scores directly correlated with pain relief on multiple measures. Minor adverse effects seen in the 10 mL group included transient diarrhea in 1 and lightheadedness in 1. Minor adverse effects seen in the 20 mL group included transient diarrhea in 5 and nausea and vomiting in 1.

### 3.2. Complications

There were minor self-limited postprocedure adverse effects in 6 subjects including lightheadedness in 1 (5%), transient diarrhea in 2 (10%), and transient nausea and vomiting in 3 (15%). Only subject in the 20 mL group required an emergency room visit for pain control at week 2. There were no complications of bleeding or paralysis.

## 4. Discussion

There were no major complications seen with the 20 mL of 98% alcohol group compared to the 10 mL group. During the EUS-CPN procedure all patients received a combination of fentanyl, versed, and propofol for sedation which likely explains the absence of pain in the recovery room. We found that EUS-CPN with 20 mL of alcohol compared to 10 mL of alcohol was safe. However, we also noted that clinical outcomes of the two groups were similar with respect to overall pain relief (100% in 20 mL group versus 80% in 10 mL group), weekly pain scores, onset of pain relief, duration of pain relief, and proportion of complete responders. This suggests that 20 mL alcohol during EUS-CPN may not provide significant pain relief despite its safety. A statistically significant difference is not observed between the two groups, however, as our study was a pilot study. A larger prospective study is warranted to confirm our findings. 

We also noted an equal number of complete responders in each group. Two thirds of these complete responders in each group had direct injection of celiac ganglia. This suggests that direct visualization and injection of celiac ganglia may improve the accuracy of EUS-CPN. The overall efficacy, however, of EUS-CPN is likely explained by the diffuse spread of alcohol in the celiac region which likely explains this result. 

With respect to pancreatic pain, digestive symptoms, nausea, and vomiting, relatively better HRQoL scores were seen in the 20 mL group. This finding has not previously been reported, and a larger prospective study is necessary for confirmation. There is a subtle trend in improved survival in the 20 mL group. Survival in general may be related to pain relief and may reflect the patient's ability to perform daily activities of living and have better nutrition. A larger prospective study, however, is necessary to confirm these observations. 

It has been reported that patients with pancreatic cancer have lower pain scores and pain medication consumption up to 16 weeks after EUS-CPN [15, 21]. In 2006 the precise identification and injection of celiac ganglia during EUS-CPN were reported in 16 of 22 (73%) patients [22]. In 2008 efficacy and safety of directly injected celiac ganglia during EUS-CPN were reported in 17 patients with unresectable pancreatic cancer and pain [16]. At 2 and 4 weeks, 94% of subjects reported pain relief as “complete,” or “partial” [16]. Likewise, percutaneous and intraoperative celiac plexus neurolysis (CPN) have been performed with varying concentrations (50% to 100%) and amounts (15 to 50 mL) of alcohol [23–27] with efficacy ranging from 70% to 90% [23]. The variable response rates may be attributed to lack of targeted injection into ganglia [5, 23, 26, 28–30]. In a recent meta-analysis of percutaneous CPN, pain relief was rated as good to excellent in 89% after 2 weeks, partial to complete in 90% at 3 months, and partial to complete in 70–90% at the time of death [23]. 

In our study minor self-limited postprocedure adverse effects were seen in a third of subjects including transient lightheadedness, diarrhea, nausea, and vomiting. Our experience is similar to previous reports of adverse effects including diarrhea (17%), postural hypotension (1%), and postprocedure-related abdominal pain (9%) [14, 31]. In a report by Levy and colleagues, 7 of 17 patients (36%) experienced pain exacerbation in the recovery room which lasted 2.2 days [16]. Procedure-related transient abdominal pain was noted in 9% of subjects who did not have direct injection of celiac ganglia [14]. There have been 2 reports of major complications occurring after EUS-CPB using steroids for chronic pancreatitis including death after an arterial pseudoaneurysm hemorrhage [32], and a peripancreatic abscess developed 5 days after EUS-CPB treated with intravenous antibiotics [33]. A recent report of acute spinal cord infarction following EUS-CPN was attributed to 24 mL of a 1 : 5 mixture of bupivacaine 0.25% with epinephrine and alcohol (5 mL into a celiac ganglia, and 19 mL into the celiac trunk area) [13]. In this patient with pancreatic adenocarcinoma of the head, the procedure resulted in paraplegia. This incident lends to support that targeted injection of the celiac ganglia is preferred, as the spinal artery can be injured during EUS-CPN.

Lillemoe and colleagues demonstrated improved survival after intraoperative CPN (20 mL of 50% alcohol) in a double-blinded randomized placebocontrolled study of 139 subjects with unresectable pancreatic cancer [5]. A survival benefit was not seen, however, with percutaneous CPN (20 mL 100% alcohol) in patients with unresectable pancreatic cancer [26]. 

The main limitation of our study is the size. In our pilot study we have demonstrated preliminary evidence that EUS-CPN 20 mL 98% alcohol appears safe. Injection of ganglia appears to improve accuracy of EUS-CPN and possibly the duration of pain relief. A larger prospective trial is warranted to confirm our findings and further understandhowto examine the potential predictors of adjuvant therapy (chemotherapy, radiation, or both) and direct ganglia injection (versus injection into the celiac region) on the clinical outcomes of pain relief, quality of life, and survival.

## Figures and Tables

**Table 1:** Patient characteristics.

	10 mL alcohol	20 mL alcohol	*P* value
Gender			0.65
Male	5	4	
Female	5	6	
Mean (SD) age	66 (14)	63 (10)	0.5
Race			0.3
Caucasian	9	10	
African American	1	0	
Location in pancreas			0.41
Head	3	4	
Uncinate		1	
Body	4	1	
Tail	0	1	
Multiple	3	3	
Immediate complications			0.11
None	8	4	
Lightheadedness	1		
Diarrhea	1	5	
Nausea/vomiting	0	1	
Ganglia injection	5	5	1
Adjuvant radiation	4	2	0.61
Adjuvant chemotherapy	6	7	0.87
ED visit prior EUS-CPN*	4	5	0.81

*Subjects who visited the emergency department for pain control prior to study entry.

**Table 2:** Characteristics of pain relief.

	10 mL alcohol	20 mL alcohol	*P* value
Total subjects	10	10	
Number of subjects who had ganglia injected	5	5	1
Pain relief	8 (80%)	10 (100%)	0.21
Median (range)* onset* of pain relief (days)	1 (1–14)	1 (1–14)	
Mean (SD) *onset* of pain relief (days)	4.3 (6.0)	4.2 (5.5)	0.81
Median (range) *duration* of pain relief (weeks)	3.0 (0–32)	3.5 (2–30)	
Mean (SD) *duration* of pain relief (weeks)	7.9 (10.8)	8.4 (9.2)	0.9

SD: standard deviation.

**Table 3:** Pain scores over time.

Time	10 mL alcohol				20 mL alcohol			
	*N *	Mean	SD	% relief*	*N *	Mean	SD	% relief*
Baseline	10	5.9	2.81		10	5.5	3.06	
24 hours	10	2.9	1.79	80%	9	3.6	3.5	67%
Week 1	8	3.75	3.28	50%	10	3.6	2.5	60%
Week 2	8	3.88	2.9	63%	8	3.13	3.27	75%
Week 3	8	3.13	2.85	75%	8	1.88	2.17	88%
Week 4	4	2	1.83	100%	5	2.2	2.17	100%
Week 5	4	2.75	0.5	100%	3	2	1.73	100%
Week 6	3	1	1.73	100%	2	2	2.83	100%

NRS: numeric rating scale (0–10), SD: standard deviation, *pain relief as defined by a 30% reduction in pain scores without an increase in pain medication consumption.

**Table 4:** Characteristics of complete responders*.

Characteristics	10 mL alcohol	20 mL alcohol
*N *	3	3
Mean age (SD)	51 (10)	66 (10)
Number of subjects with ganglia injected	2	2
Median (range) *onset* of pain relief (days)	1 ( 1–14)	1 (1–14)
Median (range) *duration* of pain relief (weeks)	23 (3–32)	8 (3–30)
Median (range) *duration* of complete response (weeks)	17 (1–22)	11 (3–21)

*Complete responders were subjects who had pain scores of zero.

**Table 5:** Quality of life scores at 4 weeks.

	10 mL alcohol			20 mL alcohol		
	*N *	mean	SD	*N *	mean	SD
Higher score is better						
Physical functioning	4	63.3	11.5	4	43.3	42.3
Role functioning	4	58.3	21.5	4	29.2	47.9
Emotional functioning	4	62.5	28.5	4	56.3	41.6
Cognitive functioning	4	50	23.6	4	45.8	45.9
Social functioning	4	62.5	21	4	58.3	41.9
Global health status/QoL	4	54.2	16	4	47.9	23.9
Lower score is better						
Fatigue	4	50	14.3	4	66.7	24
Nausea/vomiting	4	33.3	27.2	4	25	21.5
Pain	4	33.3	0	4	58.3	28.9
Dyspnea	4	16.7	33.3	4	50	43
Insomnia	4	16.7	19.2	4	25	50
Appetite loss	4	50	19.2	4	66.7	38.5
Constipation	4	16.7	19.2	4	33.3	27.2
Diarrhea	4	16.7	19.2	4	25	31.9
Financial problems	4	33.3	27.2	4	50	57.7
Higher score is better						
PANCPAIN	4	54.2	21	4	60.4	26.7
DIGSYPTOMS	4	50	19.2	4	70.8	28.5
ALTBOWELHAB	4	79.2	16	4	58.3	39.7
HEPATIC	4	80.6	5.6	4	75	19
BODYIMAGE	4	70.8	21	4	41.7	50
SATHEALTHCARE	4	83.3	19.2	4	87.5	16
SEXUALITY	4	54.2	31.5	4	50	57.7

Overall	4	62.5	9	4	59.9	17.1

SD: standard deviation, dyspnea: shortness of breath, PANCPAIN: pancreatic pain, DIGSYPTOMS: digestive symptoms, ALTBOWLHAB: altered bowel habits, HEPATIC: hepatic/liver abnormalities, BODYIMAGE: body image, SATHEALTHCA: satisfaction with health care, SEXUALITY: sexual function.
